# New Treatments for Vulvovaginal Candidiasis

**DOI:** 10.1155/S1064744996000476

**Published:** 1996

**Authors:** Sebastian Faro

**Affiliations:** Department of Obstetrics and Gynecology Rush Medical Center 1653 West Congress Parkway Room 720 Pavilion Chicago IL 60612 USA


Infectious Diseases in Obstetrics and Gynecology 4:247–254 (1996)
© 1996 Wiley-Liss, Inc.

### New Treatments for Vulvovaginal Candidiasis

**Sebastian Faro**
*Department of Obstetrics and Gynecology, Rush Medical Center, Chicago, IL*

KEY WORDS
Vulvovaginal candidiasis; *Candida;* fluconazole

Vulvovaginal candidiasis (VVC) is probably the most common form of vaginal infection, although bacterial vaginosis is believed to be more frequent.[1,2] Even though *Candida* assumes a role as a pathogen, it is an opportunistic organism causing systemic infections usually in patients who are immunosuppressed or critically ill on multiple antibiotics. However, several studies have demonstrated that *C. albicans* is a member of the host's endogenous vaginal and gastrointestinal microflora. Auger and Jolly[3] isolated *Candida* from approximately 70% of healthy women. Other investigators[4–6] have isolated *C. albicans* from the lower genital tracts of healthy, asymptomatic women with a frequency of 5–55%.

It is estimated that 75% of all women will experience one episode of symptomatic VVC during their lifetime.[7,8] Unfortunately, 40–50% of women will have at least one recurrence of VVC and at least 5% will have chronic or repeated episodes of VVC.[1]

Perhaps this condition should be viewed as a disturbance in the vaginal ecosystem rather than a disease in view of the fact that *Candida* is part of the complex endogenous microflora of many individuals. Alterations in the vaginal ecosystem can be favorable to the growth of *C. albicans* and other species. Once the yeast becomes dominant, it may result in a symptomatic condition and even produce findings consistent with acute infection. No doubt, the symptomatic condition is extremely prevalent, as evidenced by the 13 million prescriptions written in the United States between 1980 and 1990.[1]

While *C. albicans* is the predominant yeast, causing 85–90% of all cases of VVC, information is slowly being accumulated on a rising incidence of VVC due to non-*albicans* species such as *C. (Torulopsis) glabrata*, *C. tropicalis*, and *C. krusei* (Table 1).[9] This rising incidence has become even more of a concern in view of the availability of several antimycotic agents that no longer require a prescription (over-the-counter products). The easy accessibility of antimycotic intravaginal preparations has raised at least three major concerns: 1) The wanton or indiscriminate use of these agents may exert selective pressure for resistant strains; 2) the indiscriminate use of these agents may allow for replacement by other species such as *C. glabrata*, *C. tropicalis*, or *C. krusei;* and 3) the treatment may be misdirected if the individual has another or concomitant infection. Added to these concerns is the fact that the

Correspondence to: Dr. Sebastian Faro, Department of Obstetrics and Gynecology, Rush Medical Center, 1653 West Congress Parkway, Room 720 Pavilion, Chicago, IL 60612.

Review Article

TABLE 1. Species of *Candida* known to cause VVC

| Species | Incidence (%) |
|---|---|
| *C. albicans* | 90 |
| *C. glabrata* | 5 |
| *C. guilliermondi* | <1 |
| *C. krusei* | <1 |
| *C. lusitaniae* | <1 |
| *C. parapsilosis* | <1 |
| *C. pseudotropicalis* | <1 |
| *C. rugosa* | <1 |
| *C. tropicalis* | <1 |

imidazoles are less effective against non-*albicans* species.[10,11]

This trend of non-*albicans* species to occur more frequently is significant in other ways. Horowitz et al.[12,13] and MacGregor[14] reported that patients with *C. tropicalis* VVC were twice as likely to experience a recurrence than individuals infected with *C. albicans.* The incidence of non-*albicans* species has focused on *C. tropicalis* and *C. glabrata;* however, a recent increase in the isolation of *C. lusitaniae* and *C. parapsilosis* has been noted. In fact, the incidence of non-*albicans* species involved in VVC was 9.9% in the 1970s and rose to 21.3% in the 1980s.[15,16] Moreover, the role of *C. glabrata* in recurrent VVC appears to be gaining significance, causing 22.7–30.5% of the cases.[16–18] However, there seems to be a controversy over the incidence of *C. glabrata* as a causative agent for vulvovaginitis. Several investigators continue to report that *C. albicans* is responsible for 85–90% of the cases of VVC.[18a–c] To solve this growing dilemma, we should seek to answer two questions: 1) Is there a true change in colonization of the human host, namely the gastrointestinal tract which appears to be the reservoir, and the lower genital tract? 2) Is this change in *Candida* species bringing with it a resistance to the traditionally employed intravaginal agents?

### PATHOPHYSIOLOGY OF VVC

*Candida* is not believed to initiate an infection of the vulva and vagina based simply on its presence. On the contrary, the organism can easily be cultured from the lower genital tracts of 20–40% of women who have no symptoms of vaginitis.[5,14] Therefore, it is unlikely that *Candida* is introduced into the lower genital tract prior to the onset of symptoms. More likely, a change in the vulvovaginal ecology favors the growth of *Candida.*

*Candida* is a dimorphic fungus capable of growing as a typical yeast by budding or as a filamentous form by producing pseudohypha. Typically, the organism grows by forming buds attached to the mother cell or by forming blastospores or blastoconidia. The presence of germ tubes and pseudohypha, which tends to be associated with symptomatic VVC, allows the organism to adhere to the vaginal epithelium. This ability to adhere to the vaginal epithelium fulfills one requirement of the establishment of an infection. Another requirement is fulfilled when the pseudohypha invades the squamous epithelium lining the vagina. *Candida* produces a substance known as *Candida* inhibitory product (CHIP) which blocks the release of lysosomes from polymorphonuclear leukocytes, thereby interfering with the host's ability to destroy yeast cells.[14,19] Although the yeast form of *C. albicans* is able to adhere to epithelial cells, the presence of a germ tube appears to confer a greater ability to adherence.[20]

### TREATMENT OF VVC

The treatment of VVC has focused on the use of intravaginal cream or suppositories. Until recently oral agents have been reserved for recalcitrant or persistent infections. Currently, oral fluconazole, 150 mg, is available for the treatment of acute VVC. Intravaginal preparations have been favored because of their limited systemic absorption, rapid relief of symptoms, and minimal potential for side effects. The rapid relief of symptoms is thought to be a significant secondary gain of intravaginal antimycotics, especially the cream preparations. It is interesting to note that the evolution of intravaginal antimycotic agents has involved the development of shorter and shorter regimens. The treatments are administered for 3 days such as terconazole (Terazol®, Ortho Pharmaceutical Corporation, Raritan, NJ), or for 1 day, such as tioconazole (Vagistat®, Fujisawa SmithKline Corporation, Bala Cynwyd, PA) (Table 2). An algorithm for the management of acute VVC is given in Figure 1.

Prior to the introduction of fluconazole, 150 mg, oral antimycotic therapy used for the treatment of VVC has not enjoyed much popularity. Ketoconazole (Nizoral®, Janssen Pharmaceutica, Inc., Piscataway, NJ) has not been used widely except in the immunosuppressed patient or the individual with chronic, recurrent, or persistent infection because

TABLE 2. Antifungal agents for treatment of VVC

| Agent | Regimen | Route |
|---|---|---|
| Nystatin (polyene) | 14 days b.i.d. | Intravaginal |
| Butoconazole (imidazole) | 3–6 days q.d. | Intravaginal |
| Clotrimazole (imidazole) | 3–7 days q.d. | Intravaginal |
| Miconazole (imidazole) | 3–7 days q.d. | Intravaginal |
| Tioconazole (imidazole) | 1 day | Intravaginal |
| Terconazole (triazole) | 3–7 days | Intravaginal |
| Fluconazole (triazole) | 1 day | Oral |
| Ketoconazole (triazole) | 14 days | Oral |
| Itraconazole (triazole) | 1 day b.i.d. | Oral |

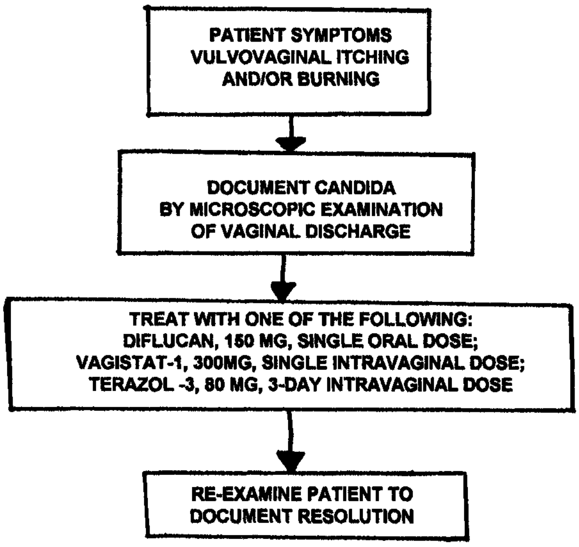

Fig. 1. Algorithm: management of acute VVC.

of its adverse effects. The most common adverse effects are nausea, vomiting, abdominal pain, inhibition of androgen biosynthesis, and hepatotoxicity.[21] Hepatotoxicity has caused a significant concern, after having been originally reported to occur in 1/10,000 ketoconazole-treated patients and later in 1/70,000. However, ketoconazole is usually prescribed for a shorter duration in patients with VVC, thus having less chance of adverse effects. Hepatotoxicity occurs more frequently in women than in men. It also occurs more frequently in patients treated for chromomycosis, previously treated with griseofulvin, or treated with ketoconazole for more than 2 weeks, as well as in patients older than 50 years.[22]

Fluconazole (Diflucan®), a new triazole, is an orally administered antifungal agent that is effective against *C. albicans* as well as *C. parapsilosis*, *C. glabrata*, and *C. tropicalis*. In vitro sensitivity studies of 384 isolates of *C. albicans* revealed 8.8% to be resistant to fluconazole; of 84 isolates of *C. tropicalis*, 34.5% were resistant; of 84 isolates of *C. glabrata*, 19.1% were resistant; and of 69 isolates of *C. parapsilosis*, 4.4% were resistant to fluconazole.[23] The imidazoles tend to be ineffective in the treatment of non-*albicans* VVC.[10]

Fluconazole offers an advantage in that a single 150-mg tablet is as effective as 3- or 7-day administration of intravaginal clotrimazole.[24] Furthermore, a single 150-mg oral dose of fluconazole is not only as effective as 3 days of intravaginal clotrimazole, but also is as effective as 5 days of oral ketoconazole or a single dose of miconazole or econazole.[25–28] There appears to be no difference with regard to recurrent disease between oral fluconazole and any of the intravaginal topical treatments.

The effectiveness of a single dose of fluconazole is most likely due to its ability to achieve significant concentrations and remain in the tissues for a prolonged time. Fluconazole concentrations in the vaginal tissues following a single 150-mg orally administered tablet were found to be 3.18 μg/g at 6 h and 0.54 μg/g at 72 h.[29] In plasma, the concentrations were 2.82 μg/nl at 6 h and 2.42 μg/nl at 72 h. Fluconazole can be detected in vaginal secretions for at least 7 days, but the concentration is slightly higher than the concentration in the vaginal tissues. In addition to persistent tissue concentrations, which may play a significant role in preventing recurrences, orally administered fluconazole combats *Candida* in the gastrointestinal tract, which is considered by some investigators to be the reservoir.[26]

Comparative studies between fluconazole and intravaginal antimycotics administered in a single course or multiple courses revealed all agents to be equivalent with regard to mycological cures.[30] Symptomatic relief was achieved in 1 day with fluconazole and in 2 days with intravaginal clotrimazole. Complete relief was achieved within 2–3 days with fluconazole and intravaginal clotrimazole, respectively.[31]

Fluconazole is similar to ketoconazole in regard to adverse effects. The most common adverse effects reported following an oral dose of fluconazole were nausea (2.1%), headache (0.9%), abdominal pain (0.7%), diarrhea (0.6%), and dyspepsia (0.6%).[30] These side effects have also been reported with ketoconazole and itraconazole. In a study of intravaginal antimycotics, 4% of the patients experienced adverse effects, namely, vaginal burning, itching, pain, and discharge.[30]

Liver abnormalities occurred in both the group receiving fluconazole (3.3%) and in the group receiving either an oral or intravaginal antimycotic (2.9%), with no significant difference between the two. These transient laboratory abnormalities were noted to be normal at the long-term follow-up examinations.[21,30,31]

A single 150-mg oral dose of fluconazole taken after the last menstrual period was not found to be associated with fetal abnormalities or an increased risk of spontaneous abortion. Pregnant women who had taken a single 150-mg dose of fluconazole after their last menses were found to have an overall rate of fetal abnormalities of 2.5%, which was not different from the 2% rate in the general population.[32,33]

Additional side effects have been reported with fluconazole but not at a significant rate. The most common of these are dizziness, dry mouth, rash, and dry skin.[8,34,35]

### DRUG INTERACTIONS

Drug interactions with intravaginal preparations are rare because of the limited absorption of these agents into the patient's bloodstream. Concern over the oral agents has been greater because of the systemic absorption of these agents from the gastrointestinal tract. Both ketoconazole and fluconazole are equipotent inhibitors of fungal cytochrome P-450 enzymes; however, fluconazole has 100 times the inhibitory activity for fungal enzymes than ketoconazole has.[36,37]

There have not been any reports of drug interactions between fluconazole and antipyrine or oral contraceptives. Fluconazole did not affect the level of estradiol nor the pharmacokinetics of ethinyl estradiol or norgestrel.[38,39]

The drugs that have been reported to interact with fluconazole are listed in Table 3. These interactions occurred in patients who were receiving high doses of fluconazole. To date, no clinically significant drug interactions have been reported following a single 150-mg dose of fluconazole.[42–50]

**TABLE 3. Drugs that interact with fluconazole**

| |
|---|
| Phenytoin |
| Warfarin |
| Cyclosporine[a] |
| Rifampin[b] |
| Zidovudine |

[a]Cyclosporine doses of >200 mg may increase serum levels of fluconazole.
[b]Lowers levels of fluconazole by up to 30%.

### RESISTANCE TO ANTIFUNGAL AGENTS

A variety of regimens has been used in the treatment of chronic VVC, ranging from a daily to a weekly or monthly administration.[53] The readily available antifungal agents, i.e., over-the-counter intravaginal azoles, have focused a great deal of attention on the potential of *Candida* to develop resistance to these agents. Also adding to this concern has been the use of these agents in a prophylactic or suppressive role in patients with chronic VVC.

It is estimated that over 20 million prescriptions, including over-the-counter usage, are written annually for intravaginal medications for the treatment of VVC. However, the use of intravaginal agents for VVC exceeds that number in view of over-the-counter agents. In spite of this overwhelming usage of antimycotics, both intravaginal and oral preparations, the development of resistance by *C. albicans* appears to be a rare event. Seven cases of *C. albicans* azole resistance have been reported, all in patients receiving high dose therapy.[54] Three of the isolates were resistant to miconazole and four to ketoconazole. There have been no reports of resistance developing with single, 7-, or 14-day therapy with intravaginal preparations. Similarly, no resistance has been noted to emerge as the result of oral single-day treatments.

### CHRONIC VVC

There is little doubt that VVC is a common condition. It inflicts a great deal of suffering on the individual, especially one who has chronic or recurrent VVC. The approach to treating chronic or recurrent VVC has been to use intravaginal or oral antimycotics in an attempt to cure the acute episode, followed by a program of maintenance therapy over a prolonged time, e.g., 4–6 months. Maintenance therapy is defined as the administration of an antimycotic once a month. The purpose of the monthly administration is to suppress the growth of *Candida*. However, the use of an agent on a monthly basis seems to be doomed to failure in view of the fact that *Candida* is part of the endogenous microflora of the vaginal ecosystem. Since *Candida* can be isolated from approximately 40% of asymptomatic women whose vaginal ecosystem may be in a balanced state, achieving therapeutic success might be better directed to reestablishing a healthy or balanced vaginal ecosystem.

It is often not possible to identify the factor(s) responsible for upsetting the delicate equilibrium of a balanced vaginal ecosystem. Nevertheless, it seems logical to try to suppress the growth of *Candida* long enough to allow the microbes that are antagonistic to the growth of the yeast to flourish. Such suppression might be accomplished with a short course of therapy, either oral or intravaginal, e.g., single-dose therapy with either fluconazole, 150-mg oral tablet, or intravaginal cream, tioconazole, 300-mg ointment. Short-course therapy may also be accomplished with a 3-day regimen of terconazole, 80 mg (see Fig. 1). Since these short-course regimens are as effective as the traditional longer therapeutic regimens, the former would probably achieve a better level of compliance. An algorithm for the management of chronic VVC is presented in Figure 2.

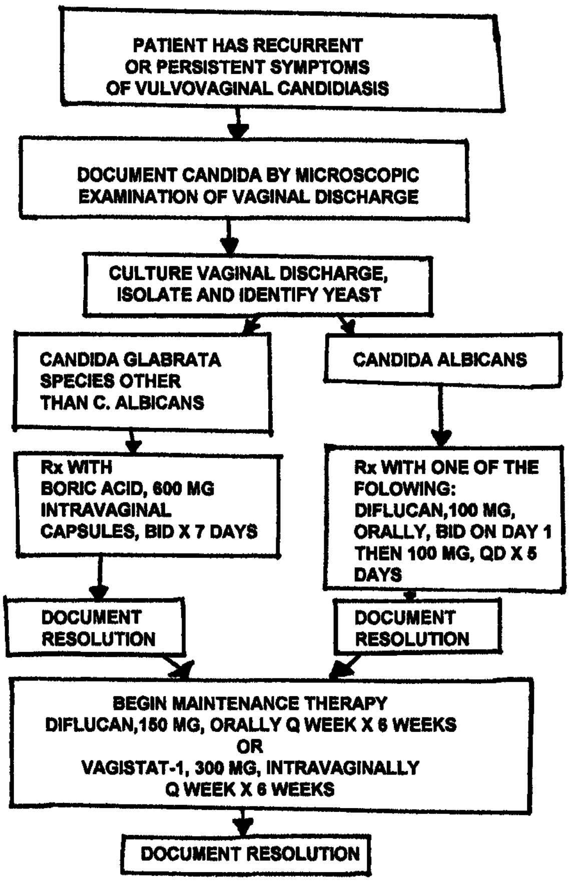

Fig. 2. Algorithm: management of chronic VVC.

### CONCLUSIONS

VVC is a common condition inflicting a great deal of suffering on the infected patient, especially if she has chronic or recurrent infections. The approach to treating this condition has been to use intravaginal or oral antimycotics in an attempt to effect a cure. However, the objective of effecting a cure may be inappropriate. Since *Candida* is part of the endogenous microflora of the vaginal ecosystem, a more logical approach would be to reestablish a healthy or balanced vaginal ecosystem.

A healthy vaginal ecosystem could be achieved with either oral or vaginal short-course therapy. Single-dose therapy could be accomplished with oral fluconazole, 150 mg, or intravaginal tioconazole, 200 mg, or a 3-day regimen of terconazole (Fig. 1). These agents are as effective as longer therapeutic regimens, so the shorter therapeutic regimen should be associated with better compliance.

The concept of treating VVC as a disturbance of the vaginal ecosystem and not a disease is supported by the high relapse rate common to all therapeutic regimens. VVC does not result from the introduction of the fungus into the vagina, but from the factor(s) that stimulates the growth of *Candida*. This growth occurs when the antagonistic effect of other microflora is overcome and, most likely, a physiologic alteration is produced that favors the growth of *Candida* while inhibiting the growth of its antagonistic microbes. Therefore, treatment directed at killing *Candida* without knowing what environmental factor(s) regulates the growth of this fungus can only result in recurrent episodes of VVC. Patients with chronic or recurrent VVC should be approached with the goal of suppressing the growth of yeast, thereby reducing the yeast population below a critical threshold to render their presence asymptomatic. This approach, by altering the environment, would result in a correction of the environmental disturbance and resolution of the chronic condition.

Prior to undertaking suppressive therapy, it is extremely important that the patient's current episode of VVC be resolved. The principle of suppressive, maintenance, or prophylactic therapy is to

TABLE 4. Factors that may contribute to VVC

| |
|---|
| Diabetes mellitus |
| Acquired immunodeficiency syndrome |
| Exogenous estrogen therapy |
| Use of oral contraceptives |
| Use of antibiotics |

prevent the growth or resurgence of *Candida*, not to eradicate the organism, cure the patient, or cure a symptomatic infection. Additionally, intrinsic or extrinsic contributing factors should be sought. Table 4 cites the factors believed to predispose a patient to VVC, although they have not been substantiated as such.[53,55]

Other possible contributing factors are rectal or gastrointestinal colonization and penile colonization of male sexual partners of women with chronic VVC. Although data exist both to support and not to support the role of gastrointestinal colonization as a contributing factor to the perpetuation of VVC, it is still an issue in patients with chronic VVC. While studies using oral nystatin did not result in a resolution of chronic VVC,[56,57] other studies of recurrent or chronic VVC have demonstrated that the *Candida* strain isolated from the patient's vagina and the strain from the rectum are identical in the majority of cases.[56,58] Therefore, until this issue is settled, the physician should not discount the possibility of gastrointestinal carriage as a contributing factor to the recurrence of VVC.

The possibility that VVC is a sexually transmitted disease has been neither substantiated nor disproved. *Candida* has been isolated from the penile skin of 20% of the male partners of women with chronic VVC.[59,60] Although asymptomatic colonization of the males occurs, it is 4 times more likely to occur in the male partners of women with recurrent VVC infections.[60] Additional support of the role of sexual transmission cannot be ruled out. For this reason, treating the male partner should be considered in the therapy of the woman with chronic VVC. If treatment of the male is contemplated, a systemic agent, such as fluconazole, should be utilized to target not only the colonization of the penile skin but also the prostate gland and gastrointestinal tract.

Evidence suggests that women with chronic or recurrent VVC had a defect in cell-mediated immunity.[63–64] A specific impairment of the T-lymphocyte is a response to *Candida* antigen, while a normal proliferative response occurs to mitogens and other non-*Candida* antigens in women with recurrent or chronic VVC.[65]

Prior to the approval of fluconazole, the prophylactic or maintenance administration of oral ketoconazole or intravaginal antimycotics reduced the frequency of recurrent episodes of VVC. The need for an oral agent became apparent because of the prolonged administration required with antimycotics. However, the available agents required daily administration. Ketoconazole was administered at an initial dose of 400 mg daily for 5 days beginning on the first day of menstruation each month for 6 months. The most effective regimen was the 100-mg daily dose for 6 months, with only 5% of the patients experiencing a recurrence.[37] Nevertheless, upon cessation of the prophylaxis, approximately 50% of the patients relapsed.

In a multicenter, international, double-blind, placebo-controlled clinical trial, the patients were given either fluconazole, 150 mg, or placebo once a month for 12 months.[66] Sixty-eight percent of the patients receiving the placebo experienced recurrences of VVC, whereas 42% of the patients receiving fluconazole had recurrences ($P = 0.02$). Patients in the placebo group experienced a 3-fold increase per patient of recurrent infection. No serious side effects were noted to have occurred.

In the European study of this international trial, the patients received 150 mg of fluconazole or placebo once a month for 4 months.[66] All patients were examined 6 months after the cessation of fluconazole prophylaxis. In this patient population, 53% of those receiving placebo experienced recurrent VVC compared with 39% of those receiving fluconazole ($P = 0.05$). Over the 6-month follow-up period, no difference was noted with regard to recurrence of VVC between the two groups.

Fluconazole offers two benefits over ketoconazole when used for therapy or prophylactic maintenance. First, fluconazole therapy is associated with less toxicity. Second, because of its long half-life, fluconazole can be given as a single dose for therapy and once a month for suppression. However, it may be more beneficial to administer prophylaxis once a week for 6–12 weeks to expose the areas colonized by *C. albicans* to a constant, not intermittent, concentration of fluconazole.

An alternative to fluconazole would be the 3-times-weekly administration of Vagistat-1 or

Terazol, leaving the choice up to the patient. Since there are no data addressing this issue, I would recommend a weekly application for 6 weeks.

### REFERENCES

1. Sobel JD: Candidal vulvovaginitis. Clin Obstet Gynecol 36:153–165, 1993.
2. Horowitz BJ, Giaquinta D, Ito S: Evolving pathogens in vulvovaginal candidiasis: Implications for patient care. J Clin Pharmacol 32:248–255, 1992.
3. Auger P, Jolly J: Microbial flora associated with *Candida albicans* vulvovaginitis. Obstet Gynecol 55:397–407, 1980.
4. Drake TE, Maibach HI: Candida and candidiasis. I. Cultural conditions, epidemiology and pathogenesis. Postgrad Med J 53:83–87, 1973.
5. Odds FC: *Candida* and Candidosis. London: Balliere-Tindall, pp 104–110, 1988.
6. Fleury FJ: Adult vaginitis. Clin Obstet Gynecol 24:407, 1981.
7. Corson SL, Kapikian RR, Nehring R: Terconazole and miconazole cream for treating vulvovaginal candidiasis. A comparison. J Reprod Med 36:561–567, 1991.
8. Osser S, Haglund A, Westrom L: Treatment of candidal vaginitis. A prospective randomized investigator-blind multicenter study comparing topically applied econazole with oral fluconazole. Acta Obstet Gynaecol Scand 70:73–78, 1991.
9. Horowitz BJ: Mycotic vulvovaginitis: A broad overview. Am J Obstet Gynecol 165:1188–1192, 1991.
10. Redondo-Lopez V, Lynch M, Schmitt C, Cook R, Sobel JD: *Torulopsis glabrata* vaginitis: Clinical aspects and susceptibility to antifungal agents. Obstet Gynecol 76:651–655, 1990.
11. Boag FC, Houang ET, Westrom R, McCormack SM, Lawrence AG: Comparison of vaginal flora after treatment with a clotrimazole 500 mg vaginal pessary or a fluconazole 150 mg capsule for vaginal candidosis. Genitourin Med 67:232–234, 1991.
12. Horowitz BJ: The role of non-*albicans Candida* in vulvovaginal candidiasis. J Clin Pract Sexuality 7:16–20, 1991.
13. Horowitz BJ, Edelstein SW, Lippman L: *Candida tropicalis* vulvovaginitis. Obstet Gynecol 66:229–232, 1985.
14. McGregor JA: Pathophysiology of vulvovaginal candidiasis. J Clin Pract Sexuality 7:6–9, 1991.
15. Cavenbaugh G: Vaginal candidiasis: Evolving trends in the incidence and treatment of non-*Candida albicans* infections. Curr Probl Obstet Gynecol Fertil 8:241–245, 1990.
16. Takada M, Kubota T, Hogaki M, et al.: Attributes of microorganisms that contribute to recurrence and interactability of vaginal mycosis. Acta Obstet Gynecol J 38: 1125–1134, 1986.
17. Kurvabara N, Sugiura K: Treatment of intractable vaginal mycosis. Obstet Gynecol Ther 41:1, 1980.
18. Goldacre MJ, Milone LJ, Watt B, et al.: Prevalence of yeast and fungi other than *Candida albicans* in the vagina of normal young women. Br J Obstet Gynaecol 88:596–600, 1981.

18a. Sobel JD: Vaginal infections in adult women. Med Clin North Am 74:1573–1602, 1990.

18b. Faro S. Vaginitis: Diagnosis and management. Int J Fertil 41:115–123, 1996.

18c. Nyirjesy P, Seeney SM, Grody MHT, et al.: Chronic fungal vaginitis: The value of cultures. Am J Obstet Gynecol 173:S20–23, 1995.

19. Somail EH, Kolotila MP, Ruggeri R, Diamond RD: Natural inhibitor from *Candida albicans* blocks release of azurophil and specific granule contents by chemotactic peptide stimulated human neutrophils. Infect Immunol 57:689–692, 1989.
20. Sobel JD, Myers PG, Kaye D, Levison ME: Adherence of *C. albicans* to human vaginal and buccal epithelial cells. J Infect Dis 143:76–82, 1981.
21. Hay RJ: Risk/benefit ratio of modern antifungal therapy: Focus on hepatic reactions. J Am Acad Dermatol 29:550–554, 1993.
22. Fong IW: The value of chronic suppressive therapy with itraconazole versus clotrimazole in women with recurrent vaginal candidiasis. Genitourin Med 68:374–377, 1992.
23. Arévalo MP, Arias A, Andrew A, et al.: Fluconazole, itraconazole and ketoconazole in vitro activity against *Candida* spp. J Chemother 6:226–229, 1996.
24. Sobel JD, Brooker D, Stein GE, et al.: The Fluconazole Vaginitis Study Group: Single oral dose fluconazole compared with conventional clotrimazole topical therapy of *Candida vaginitis.* Am J Obstet Gynecol 172: 1263–1268, 1995.
25. Anderson GM, Barrot J, Bergan T, Brammer KW, Cohen J, Dellenbach P: A comparison of single-dose oral fluconazole with a 3-day intravaginal clotrimazole in the treatment of vaginal candidiasis. Br J Obstet Gynaecol 96:226–232, 1989.
26. Kutzer E, Oittmer R, Leodolter S, Brammer KW: A comparison of fluconazole and ketoconazole in the oral treatment of vaginal candidiasis: Report of a double-blind multicenter trial. Eur J Obstet Gynaecol Reprod Biol 29:305–313, 1988.
27. Van Heusdan AM, Corbey RS, et al.: Single dose oral fluconazole versus single dose topical miconazole for the treatment of acute vulvovaginal candidosis. Acta Obstet Gynaecol Scand 69:417–422, 1990.
28. Westrom L, Haglund A, Swensson L: Fluconazole versus econazole in treating vaginal candidiasis. Int J Gynaecol Obstet 37:29–31, 1992.
29. Houang ET, Chappatte O, Byrne D, Macrae PV, Thorpe JE: Fluconazole levels in plasma and vaginal secretions of patients after a 150 mg single dose and rate of infection in vaginal candidiasis. Antimicrob Agents Chemother 34:909–912, 1990.
30. de los Reyes C, Edelman DE, De Bruin MF: Clinical experience with single-dose fluconazole in vaginal candidiasis. A review of the worldwide database. Int J Gynaecol Obstet 37:9–15, 1992.

31. Del Palacio A: Fungal skin and soft tissue infections. Curr Opin Infect Dis 5:687–694, 1992.
32. Inman WHW, Pearce G, Wilton L: Safety of fluconazole in the treatment of vaginal candidiasis: A prescription-event monitoring study, with special reference to the outcome of pregnancy. Eur J Clin Pharmacol 46:115–118, 1994.
33. Rubin PC, Wilton LV, Inman WHW: Fluconazole and pregnancy: Results of a prescription event-monitoring study. Int J Gynaecol Obstet 37:25–27, 1992.
34. Stein GE, Christensen S, Mummaw N: Comparative study of fluconazole and clotrimazole in the treatment of vulvovaginal candidiasis. DICP 25:582–585, 1991.
35. Brammer KW, Fezzko JM: Single-dose oral fluconazole in the treatment of vaginal candidiasis. Ann NY Acad Sci 554:561–563, 1988.
36. Perfect JR, Lindsay MH, Drew RH: Adverse drug reactions to systemic antifungals. Prevention and management. Drug Saf 7:323–363, 1992.
37. Shaw JTB, Tarbitt MH, Troke PF: Cytochrome P-450 Mediated Sterol Synthesis and Metabolism: Differences in Sensitivity to Fluconazole and Other Azoles. Barcelona: JR Prous Science Publishers, 1987.
38. Grant SM, Clissold SP: Fluconazole: A review of its pharmacodynamic and pharmacokinetic properties and therapeutic potential in superficial and systemic mycosis. Drugs 39:872–916, 1990.
39. Lazar JD, Wilner KD: Drug interactions with fluconazole. Rev Infect Dis 3(Suppl):S327–S333, 1990.
40. Cadle RM, Zenon GJ III, Rodriguez-Barradas MC, et al.: Fluconazole induced symptomatic phenytoin toxicity. Ann Pharmacother 28:191–195, 1994.
41. Touchette MA, Chandraschai PH, Milad MA, et al.: Contrasting effects of fluconazole and ketoconazole on phenytoin and testosterone disposition in man. Br J Clin Pharmacol 34:75–78, 1992.
42. Blum RA, Wilton JH, Hilligoss DM, et al.: Effect of fluconazole on the disposition of phenytoin. Clin Pharmacol Ther 49:424–425, 1991.
43. Howitt KM, Oziemski MA: Phenytoin toxicity induced by fluconazole. Med J Aust 51:603–604, 1989.
44. Seaton TL, Celam CL, Black DJ: Possible potentiation of warfarin by fluconazole DICP 24:1177–1178, 1990.
45. Gerike KR: Possible interaction between warfarin and fluconazole. Pharmacotherapy 13:508–509, 1993.
46. Lopez-Gil JA: Fluconazole-cyclosporine interaction: A dose dependent effect? Ann Pharmacother 27:427–430, 1993.
47. Chan GL, Sinnott JT, Emmanuel PJ, et al.: Drug interactions with cyclosporine: Focus on antimicrobial agents. Clin Transpl 6:141–153, 1992.
48. Back DJ, Tya JF: Comparative effects of the antimycotic drugs ketoconazole, fluconazole, itraconazole and terbinafine on the metabolism of cyclosporine by human liver microsomes. Br J Clin Pharmacol 32:624–626, 1991.
49. Graves NM, Matas AJ, Hilligoss DM, et al.: Fluconazole/cyclosporine interaction (abstract). Clin Pharmacol Ther 47:208, 1990.
50. Carleton BC, Graves NM, Matas AJ, et al.: Managing the fluconazole and cyclosporine interaction: Results of a double-blind randomized pharmacokinetic and safety study (abstract). Pharmacotherapy 10:210, 1990.
51. Apseloff G, Hilligoss DM, Gardner MJ, et al.: Induction of fluconazole metabolism by infants in in vivo study in humans. J Clin Pharmacol 31:358–361, 1991.
52. Sahai J, Gallicano K, Pakuts A, et al.: Effect of fluconazole on zidovudine pharmacokinetics in patients infected with human immunodeficiency virus. J Infect Dis 169:1103–1107, 1994.
53. Sobel JD: Recurrent vulvovaginal candidiasis. A prospective study of the efficacy of maintenance ketoconazole therapy. N Engl J Med 315:1455, 1986.
54. Sobel JD: Antimycotic azole resistance in vulvovaginal candidiasis. Int J Gynaecol Oncol 37:39–43, 1992.
55. Sobel JD: Management of recurrent vulvovaginal candidosis with intermittent ketoconazole prophylaxis. Obstet Gynecol 65:435, 1985.
56. Milme JD, Warnock DW: Effect of simultaneous oral and vaginal treatment on the rate of cure and relapse in vaginal candidosis. Br J Vener Dis 56:362, 1979.
57. O'Connor MJ, Sobel JD: Epidemiology of recurrent vulvovaginal candidosis: Identification and strain differentiation of *Candida albicans*. J Infect Dis 154:358, 1986.
58. Meinhoff WL: Demonstration of typical features of individual *Candida albicans* strains as a means of studying sources of infection. Chemother 28(Suppl 1):51, 1982.
59. Davidson F: Yeasts and circumcision in the male. Br J Vener Dis 53:121, 1977.
60. Rodin P, Kolator B: Carriage of yeast on the penis. Br Med J 1:1123, 1976.
61. Hobbs JR, Bugden D, Davidson F, Kahan M, Oates JK: Immunological aspects of candidal vaginitis. Proc R Soc Med 70(Suppl 4):1, 1977.
62. Mathur S, Melcher JT III, Ades EW, Williamson HD, Fudenberg HH: Anti-ovaria and anti-lymphocyte antibodies in patients with chronic vaginal candidosis. J Reprod Immunol 2:247, 1980.
63. Syverson RE, Buckley HR, Gibian J, Ryan GM Jr: Cellular and humoral immune status in women with chronic *Candida* vaginitis. Am J Obstet Gynecol 123:624, 1979.
64. Witkin SS, Yu IR, Ledger WJ: Inhibition of *Candida albicans* induced lymphocyte proliferation by lymphocytes and sera from women with recurrent vaginitis. Am J Obstet Gynecol 147:809, 1983.
65. Witkin SS: Inhibition of *Candida*-induced lymphocyte proliferation by antibody to *Candida albicans*. Obstet Gynecol 68:696, 1986.
66. Sobel JD: Fluconazole maintenance therapy in recurrent vulvovaginal candidiasis. Int J Gynaecol Obstet 37(Suppl 1):17, 1992.
